# RNA-directed DNA methylation as a weapon in parental conflict

**DOI:** 10.1093/plphys/kiad664

**Published:** 2023-12-14

**Authors:** Dechang Cao

**Affiliations:** Assistant Features Editor, Plant Physiology, American Society of Plant Biologists; Germplasm Bank of Wild Species, Kunming Institute of Botany, Chinese Academy of Sciences, Kunming, Yunnan 650201, China

During sexual reproduction in angiosperms and mammals, the developing zygote receives genetic information from both the female and male parents. For several decades it has been apparent that some genes are differentially expressed depending on whether they are inherited from the female or male parent, which is referred to as imprinting. This imprinting is often a consequence of parental conflicts, which are due to distinct evolutionary interests between the male and female parent (Parker 1979).

In plants, parental conflicts have been recognized during seed development as a competition over resource allocation into the developing seeds (Doll and Ingram 2022). There is intense competition among seeds for resources from the mother plant, and the competition can be even more intense when the offspring come from multiple pollen donors (Bawa 2016). Specifically, the best outcome for the mother is that all the seeds receive adequate nutrients during their development. By contrast, if the seeds are offspring of different pollen donors, they compete with and conflict with their half-siblings.

Genetic studies show that some genes promoting seed development are suppressed in the female genome and enhanced in the male genome, reflecting their “equal to all” versus “most to mine” pressures. The parental conflict has led to an endless “arms race” between males and females and might have contributed to the rapid radiation of angiosperms (Doll and Ingram 2022). The proper balance of parental genomes is crucial to avoid developmental disorder and seed abortion (Lafon Placette 2020). In this issue of *Plant Physiology*, Dew-Budd et al. (2023) showed that maternal RNA-directed DNA methylation (RdDM) functions as an efficient approach to balance parental genomes in the outbreeding plant *Capsella grandiflora* (Figure 1).

**Figure 1.**
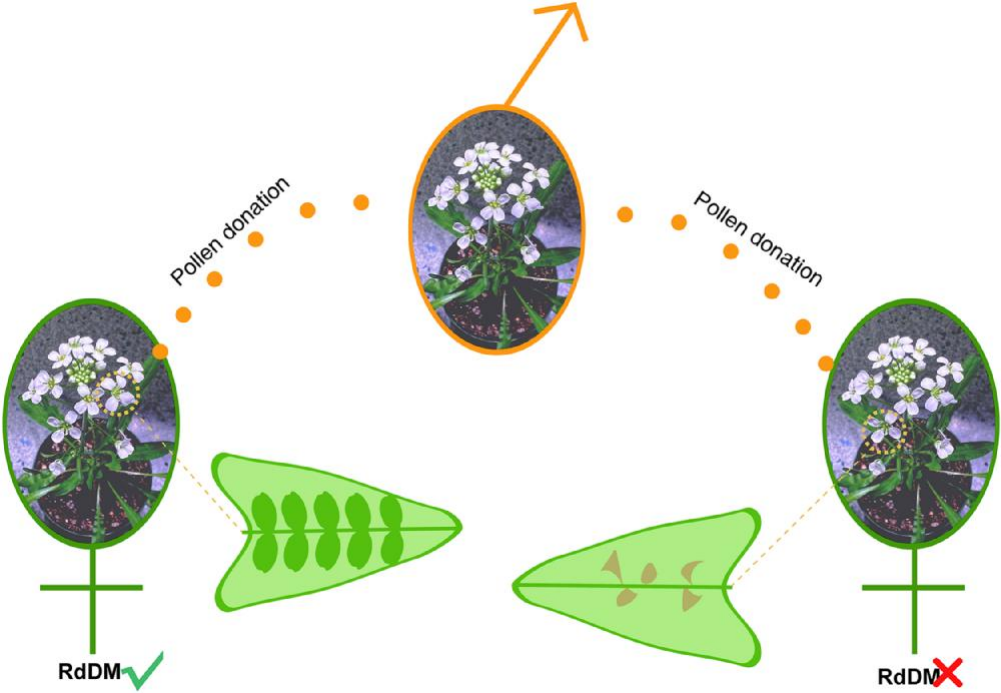
Maternal RdDM function is required for seed development in the outbreeding *Capsella grandiflora* (Brassiaceae). A deficient mutation of maternal RdDM results in seed abortion in *C. grandiflora*, and pollination with wild-type pollen cannot rescue the seed development defect. Even mutant embryos (outcrossing a heterozygous mutant plant with homozygous mutant pollen) can develop normally on RdDM-functioning mother plants. The photo of *C. grandiflora* was kindly provided by Ms. Kathryn Panferov.

Previous studies have demonstrated that small RNAs have a role in genomic imprinting and parental conflict. Work in the authors’ laboratory previously found that some 24-nucleotide (nt) small interfering RNAs (siRNAs) move to the ovule from surrounding maternal tissues (Grover et al. 2020; Burgess et al. 2022). These 24-nt siRNAs are produced in an RNA polymerase IV- and RNA-DEPENDENT RNA POLYMERASE 2 (RDR2)-dependent way [see review by Chow and Mosher (2023)] and thus are sometimes termed p4-siRNAs. P4-siRNAs mediate RdDM, which causes de novo DNA methylation to cytosines regardless of the sequence context. Mutation of RdDM has been reported to result in reproductive defects in some plants; however, the impact of RdDM is not consistent among species. Mutants of *Brassica rapa* deficient in RdDM showed severe seed abortion, while a subtle effect was observed in seed development of Arabidopsis with a RdDM mutation (Grover et al. 2018). Previously, it was proposed that intensity of parental conflict is associated with the extent of outcrossing (Brandvain and Haig 2005). Considering the difference in how self-compatible Arabidopsis and outbreeding *B. rapa* are impacted by RdDM mutation, it is likely that the importance of maternal RdDM pathway in seed development varies in different mating systems. Dew-Budd et al. (2023) tested this hypothesis using Brassicaceae species.

Mutants deficient in RdDM were generated using the CRISPR/Cas9 technology in 3 Brassicaceae species: *Camelina sativa*, *Capsella rubella*, and *Capsella grandiflora* (Dew-Budd et al. 2023). A considerable decrease in the abundance of 23-24-nt siRNAs was observed in *rdr2* lines of all 3 species, suggesting the successful interruption of the RdDM pathway (Dew-Budd et al. 2023). A slight decrease in 23-24-nt siRNAs also appeared in *nrpe1* plants, which was not beyond the expectation, because the failure of target methylation results in decreased production of siRNAs with a feedback regulation (Zhao and Chen 2014). A ShortStack pipeline was applied to evaluate the siRNA-producing loci throughout the genomes, and 80% to 90% of siRNA loci showed reduced siRNA accumulation in the *rdr2* mutants. These changes suggested substantial disruption of the RdDM pathway in the mutant plants.

Strikingly, all the *rdr2* mutants showed reduced seed set, with degrees of reduction varying in the 3 species (Dew-Budd et al. 2023). The outbreeder *C. grandiflora* showed the severest decline of 97% to 99% in the *rdr2* mutants, *C. rubella* showed a decline of about half, and *C. sativa* had 25% to 32% reduction. Generally, the *nrpe1* mutants showed relatively milder decline in seed production, and no change was observed in seed set of 1 *Cs nrpe1* mutant compared with the wild type (Dew-Budd et al. 2023). Seed weight was also significantly (*P* < 0.01) reduced in the *rdr2* and *nrpe1* mutants, with the highest reduction in *C. grandiflora* and no significant change in the *Cs nrpe1* plants.

A closer examination into the 2 *Capsella* species was further carried out to determine if the seed development defect of the RdDM mutants was attributed to maternal factors. The mature pollen was examined and pollen viability tested via fluorescein diacetate staining, which showed no detectable defects in pollen development of the RdDM mutants (Dew-Budd et al. 2023). An effort to pollinate the *Capsella* RdDM mutants with wild-type pollen did not rescue the seed development defect. Thus, seed development defect of the *Capsella* RdDM mutants should be attributed to maternal factors as either a sporophytic or gametophytic defect.

To further distinguish between the 2 types of maternal defects, Dew-Budd et al. (2023) calculated the ovule numbers and found around 25% fewer ovules in the *C. rubella* RdDM mutants and no significant changes between the *C. grandiflora* RdDM mutants and the wild type. Although there were no changes in ovule numbers, the *C. grandiflora* RdDM mutants had a considerable amount (14%-56%) of seed abortion after fertilization, leading to the seed set defect. Another interesting experiment was performed by crossing heterozygous *C. grandiflora* mutants (capable of RdDM) with wild-type or homozygous mutant pollen donors. It was found that the mutant embryo sacs developed equally well as the wild type when they are supported by an RdDM-functioning maternal sporophyte.

Taken together, these results show that the maternal RdDM function is required for seed production of the outbreeder *C. grandiflora*, whereas dysfunction of maternal RdDM did not contribute to seed development defects in the inbreeding *C. rubella* and *C. sativa*, suggesting that RdDM has a greater effect on the outbreeding species. The results agree with previous findings that the RdDM mutation leads to seed abortion in the outbreeder *B. rapa* but has no overt effect on seed production in the self-compatible Arabidopsis (Grover et al. 2018).

The results of this study suggest that it is possible that maternal RdDM takes a bigger role in balancing parental genomes for outbreeders, whose parental conflicts are more intense than in self-compatible species. More cases are needed to decide whether it is a universal mechanism across the angiosperms. However, these findings do shed new light on differential roles of RdDM function in species with various degrees of parental conflicts. Previous studies have demonstrated that paternal siRNAs move from the tapetum to the microspores and epigenetically modify the sperm cells (Long et al. 2021). The new evidence suggested that maternally derived siRNAs subsequently “fight back” by making their own modifications on zygotic DNA.

## Dive Curated Terms

The following phenotypic, genotypic, and functional terms are of significance to the work described in this paper:

RDR2 Gramene: AT4G11130
RDR2 Araport: AT4G11130

seed AmiGo: PO:0009010

seed weight Planteome: TO:0000181

NRPE1 Gramene: AT2G40030
NRPE1 Araport: AT2G40030
